# Case Report: Austrian syndrome with multifocal septic arthritis in the immunocompetent elderly: expanding the clinical spectrum

**DOI:** 10.3389/fmed.2025.1706287

**Published:** 2026-01-27

**Authors:** Sahar Zelfani, Helmi Ernandes, Salma Kaoual, Ameni Bellaaj, Sahar Sallem, Asma Ghariani, Ikbel Kooli

**Affiliations:** 1 Infectious Diseases Department, Mohamed Kassab Institute of Orthopedics, Tunis, Tunisia; 2 Laboratory of Medical Biology, Mohamed Kassab Institute of Orthopedics, Tunis, Tunisia

**Keywords:** Austrian syndrome, endocarditis, meningoencephalitis, septic arthritis, *Streptococcus pneumoniae*

## Abstract

**Introduction:**

Austrian syndrome, defined by the triad of pneumococcal pneumonia, meningitis, and infective endocarditis (IE), represents a rare but severe form of invasive pneumococcal disease (IPD). Despite advances in vaccination and antimicrobial therapy, it remains associated with high morbidity and mortality due to diagnostic delays. We report a diagnostically challenging case of Austrian syndrome in an immunocompetent elderly male, complicated by multifocal septic arthritis and delayed recognition of IE.

**Case description:**

A 71-year-old man with no history of pneumococcal vaccination, smoking, or alcohol use initially presented with acute monoarthritis of the right knee. Both blood and synovial cultures yielded *Streptococcus pneumoniae* serotype 19A with reduced susceptibility to penicillin. During hospitalization, he developed acute confusion and right wrist arthritis. Chest imaging revealed right lower lobe consolidation, and brain magnetic resonance imaging demonstrated leptomeningeal enhancement consistent with meningitis. Transthoracic echocardiography was initially negative. However, subsequent transoesophageal echocardiography revealed aortic valve vegetations with annular abscess, confirming definite IE according to modified Duke criteria. The combination of pneumonia, meningitis, and IE established the diagnosis of Austrian syndrome. The patient was managed with prolonged targeted intravenous antibiotics, achieving full clinical and microbiological recovery without surgical intervention.

**Conclusion:**

This case illustrates the diagnostic complexity of Austrian syndrome in an immunocompetent elderly patient lacking classical risk factors or respiratory symptoms. It underscores the pivotal role of transoesophageal echocardiography in IE diagnosis, highlights the therapeutic potential of prolonged targeted antimicrobial therapy, and reinforces the importance of pneumococcal vaccination to prevent severe IPD.

## Introduction

*Streptococcus pneumoniae* is an encapsulated Gram-positive diplococcus that commonly colonizes the nasopharynx and can invade sterile body sites under conducive conditions, leading to systemic dissemination (1). While pneumococcal meningitis and pneumonia are well-recognized clinical syndromes, pneumococcal endocarditis is an uncommon but life-threatening complication, particularly when it involves the aortic valve and leads to rapid valve destruction or perivalvular abscess formation (2). Austrian syndrome, also referred to as Osler's triad, is a rare but severe clinical entity characterized by the concurrent manifestation of pneumococcal pneumonia, meningitis, and infective endocarditis (IE). First described by Sir William Osler in the early 20th century and later named after Robert Austrian for his pivotal work on pneumococcal infections, this syndrome predominantly affects individuals with predisposing factors such as advanced age, immunosuppression, alcoholism, or underlying cardiac disease (3). Despite the widespread use of pneumococcal vaccines and advances in antimicrobial therapy, Austrian syndrome remains associated with high morbidity and mortality due to its fulminant course and diagnostic complexity (4). The clinical recognition of Austrian syndrome is often delayed due to its nonspecific and overlapping symptomatology, compounded by the evolving presentations of systemic sepsis and neurological impairment. Prompt identification and aggressive multidisciplinary management including early surgical intervention and targeted antimicrobial therapy are critical to improving patient outcomes (5).

We report a diagnostically challenging and clinically severe case of Austrian syndrome in an immunocompetent elderly male, with no history of pneumococcal vaccination, who developed multifocal invasive pneumococcal disease (IPD) complicated by septic arthritis, meningoencephalitis, and aortic valve endocarditis with annular abscess. This case underscores the importance of early suspicion, comprehensive diagnostic evaluation, and adherence to evidence-based management protocols in rare but high-stakes infectious syndromes, even in the absence of classical immunosuppressive risk factors.

## Case description

A 71-year-old male with complete functional dependency in activities of daily living presented with acute monoarthritis of the right knee. His medical history was significant for a decade-long course of well-controlled hypertension under angiotensin-converting enzyme inhibitor therapy. He had no history of pneumococcal vaccination, smoking, or alcohol use.

Two weeks prior to admission, the patient reported a self-limited episode of respiratory symptoms for which he was administered intramuscular dexamethasone sodium phosphate. Three days prior to presentation, he developed acute right knee pain prompting evaluation by a rheumatologist.

On physical examination, the right knee exhibited erythema, edema, local warmth, and tenderness, accompanied by significant restriction in active and passive range of motion. Arthrocentesis yielded turbid, straw-colored synovial fluid, raising suspicion for septic arthritis. Cytology demonstrated a leukocyte count of 80,000 cells/mm3, with 90% polymorphonuclear neutrophils. Gram stain was negative for microorganisms. The clinical context of acute monoarthritis with purulent synovial fluid favored a bacterial etiology, most likely *Staphylococcus aureus* or *Neisseria gonorrhoeae*, over crystal-induced or inflammatory arthritis. The patient was referred to orthopedic assessment and subsequently hospitalized.

At admission, he was afebrile but developed pyrexia (39.0 °C) within hours. Vital signs revealed hypertension (140/70 mmHg), tachycardia (110 bpm), and normoxia on ambient air (peripheral oxygen saturation: 96%). Neurological evaluation demonstrated a Glasgow Coma Scale (GCS) score of 15/15 with intact higher cognitive functions and no signs of meningeal irritation. Cardiopulmonary examination revealed fine bibasal rales and a systolic murmur at the aortic area. No evidence of trauma, dermal breaches, or recent invasive procedures were observed.

**Figure 1:**
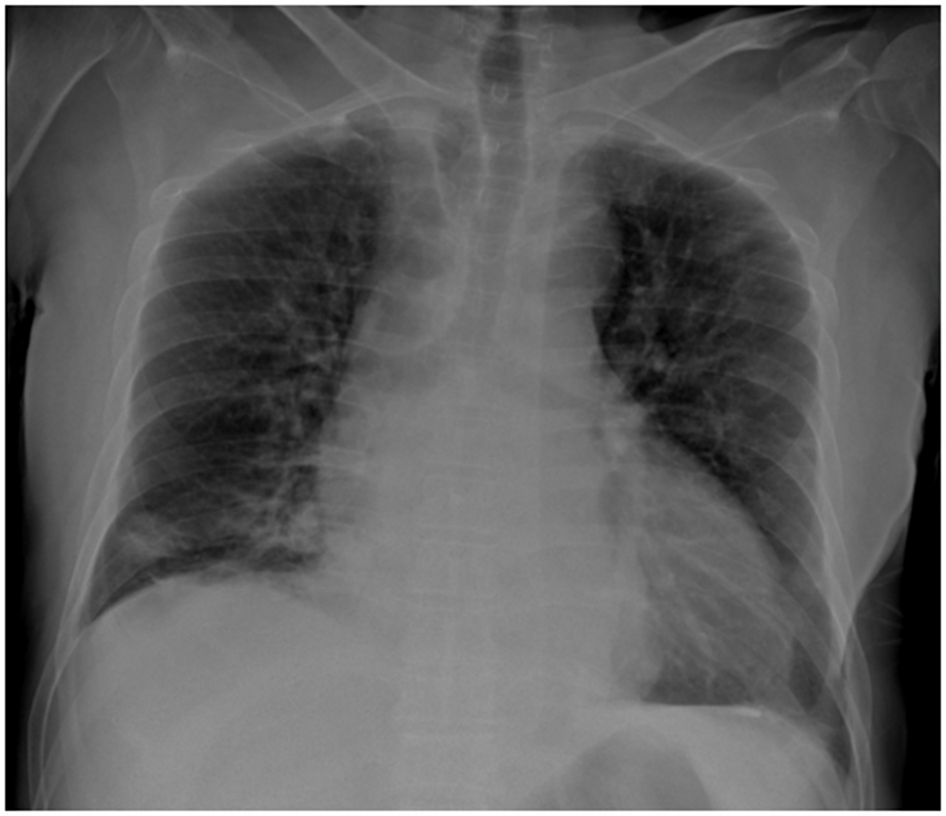
Posteroanterior chest radiograph demonstrating cardiomegaly and right basal alveolar opacities. The pleural recesses appear clear, with no evidence of pleural or parietal lesions.

Initial laboratory investigations demonstrated leukocytosis (WBC: 10,770/μl), markedly elevated C-reactive protein (CRP: 358 mg/L), and impaired renal function (eGFR: 57.86 ml/min/1.73 m^2^). Hepatic and electrolyte panels were within normal limits. Blood cultures were performed. Radiographic imaging of the right knee was unremarkable. Chest radiography revealed right lower lobe consolidation consistent with pneumonia (Figure 1). The patient underwent right knee arthrotomy with intraoperative sampling, and empirical intravenous cefazolin (2 g every 6 h) was initiated.

**Figure 2:**
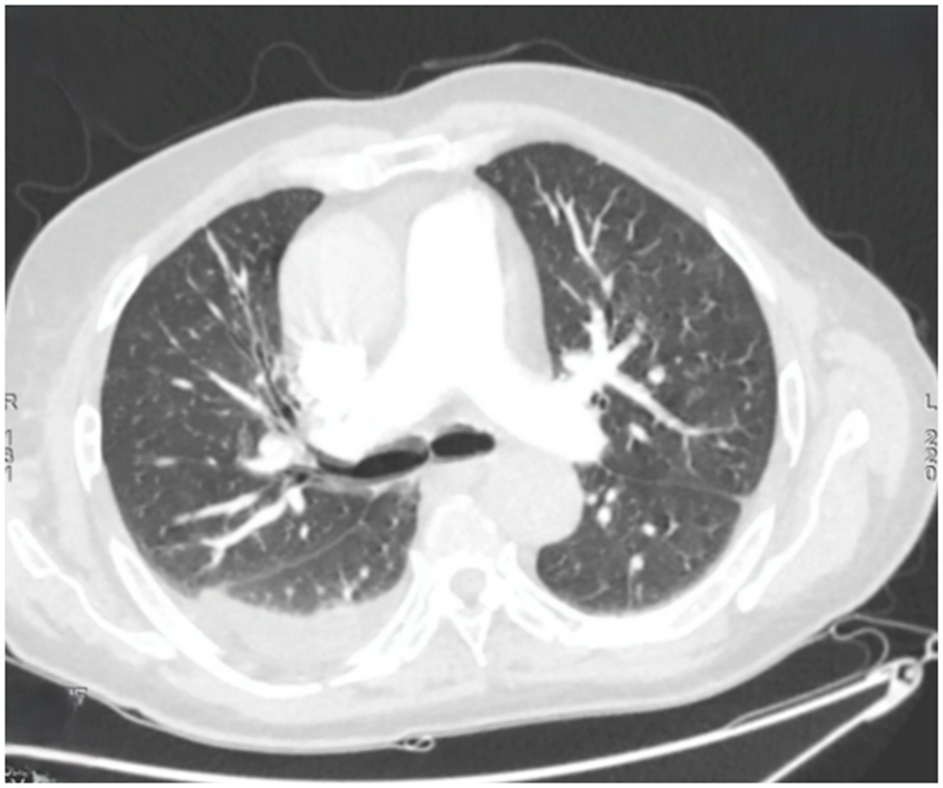
Axial chest computed tomography showing multiple bilateral ground-glass nodules, predominantly in the left upper lobe. Bilateral band-like subpleural opacities suggestive of ventilatory abnormalities are noted. A posterior basal consolidation is present in the right lung. Moderate right-sided and mild left-sided pleural effusions are identified, each associated with ipsilateral compressive atelectasis.

Within 48 h, the patient experienced acute respiratory distress characterized by tachypnea (respiratory rate: 30/min), use of accessory muscles, and oxygen desaturation to 89% on room air. The hypoxemia resolved promptly with low-flow oxygen therapy at 2 L/min via nasal cannula, permitting discontinuation of supplemental oxygen within 24 h. Thoracic computed tomography (CT) demonstrated right posterobasal consolidation and bilateral ground-glass opacities, with no evidence of pulmonary embolism (Figure 2).

Neurologically, he developed acute confusional state (GCS: 13/15) with temporal and spatial disorientation, although no focal deficits or meningeal signs were detected. At that stage, metabolic or toxic encephalopathy, septic encephalopathy, and acute ischemic stroke were considered plausible causes, warranting cerebral imaging. Non-contrast cerebral CT revealed only mild cortical atrophy. Brain magnetic resonance imaging (MRI) revealed leptomeningeal enhancement along the sulcal contours on post-contrast T1-weighted and FLAIR sequences, indicative of pia–arachnoid inflammation. Sulcal FLAIR hyperintensity within the subarachnoid space further supported the diagnosis of meningitis. Two lumbar punctures yielded dry taps, precluding cerebrospinal fluid analysis. Based on the clinical context, meningoencephalitis was presumed, and intravenous vancomycin (40 mg/kg/day) was added to the antimicrobial regimen. Adjunctive dexamethasone was withheld due to both timing and patient-specific clinical factors. Guidelines recommend corticosteroids only when administered prior to, or concomitantly with, the first dose of antibiotics, as delayed administration lacks demonstrated efficacy and may compromise antimicrobial penetration into the central nervous system (CNS). Additionally, the patient's advanced age and ongoing systemic inflammation conferred an elevated risk of corticosteroid-associated adverse effects such as dysglycemia, secondary infections, and delayed recovery, thereby outweighing any uncertain therapeutic benefit.

A secondary focus of inflammation was noted in the right wrist, characterized by swelling, erythema, and restricted mobility. Ultrasonography confirmed synovitis and tenosynovitis. The patient underwent surgical drainage of the wrist joint, where purulent material was observed. Intraoperative samples cultures yielded no microbial growth.

Given the clinical constellation of septic arthritis, pneumonia, and presumed meningoencephalitis, IE was suspected. Transthoracic echocardiogram (TTE) demonstrated preserved ejection fraction (65%), minimal valvular regurgitations, and no vegetations. Microbiological analysis of blood cultures and synovial fluid samples, including knee arthrocentesis and the initial intraoperative specimen, yielded *Streptococcus pneumoniae* exhibiting reduced susceptibility to penicillin. Serotyping of the isolates was performed using the Quellung reaction (Statens Serum Institut, Copenhagen) and confirmed as serotype 19A in both the blood and synovial fluid cultures, indicating a single invasive clone. A pneumococcal urinary antigen test was not performed. Based on the 2023 European Society of Cardiology (ESC)-modified Duke criteria, the patient met one major criterion (≥2 positive blood cultures for *S. pneumoniae*) and two minor criteria (fever >38 °C, and septic embolic arthritis), supporting a possible diagnosis of pneumococcal IE. Antimicrobial therapy was adjusted to include cefotaxime, selected over ceftriaxone due to institutional formulary availability, at a meningeal dosing regimen of 300 mg/kg/day and gentamicin at a dosage of 3 mg/kg/day.

On day seven, the patient remained febrile but hemodynamically stable. The electrocardiography was unremarkable. The surgical wound at the wrist showed ongoing bleeding, prompting coagulation studies. Laboratory findings were notable for anemia (hemoglobin: 6.5 g/dl), marked thrombocytosis (1,149,000/mm3), elevated CRP (68 mg/L), and coagulopathy (PT: 52%, aPTT: 1.6 × control), suggestive of early disseminated intravascular coagulation (DIC). D-dimer levels were elevated (2,250 ng/ml), while fibrinogen remained within normal range.

A transoesophageal echocardiogram (TEE) revealed a highly mobile echogenic mass on the aortic valve consistent with vegetation, moderate central aortic regurgitation, an aortic annular abscess, and aortic root dilatation (40 mm). These findings fulfilled two major 2023 ESC-modified Duke criteria (≥2 positive blood cultures for *S. pneumoniae*, imaging positive for IE), establishing a definite diagnosis of aortic valve endocarditis complicated by annular abscess and structural valve damage. Based on the triad of pneumococcal pneumonia, meningitis, and endocarditis, the patient was diagnosed with Austrian syndrome.

Given that IPD rarely occurs in immunocompetent hosts, an immunodeficiency workup including human immunodeficiency virus (HIV) serology, diabetes screening, and quantitative immunoglobulin levels was conducted, all of which were unremarkable.

Following a 2-week course of cefotaxime combined with gentamicin, antimicrobial therapy was adjusted to cefotaxime (150 mg/kg/day) and vancomycin (40 mg/kg/day) with discontinuation of gentamicin. The cefotaxime dose was reduced from 300 to 150 mg/kg/day, as the initial high-dose regimen provided adequate meningeal coverage, whereas the lower dose was considered sufficient for the treatment of endocarditis and septic arthritis. Vancomycin was introduced considering the infection's severity, the presence of an aortic annular abscess, and the reduced penicillin susceptibility of the pneumococcal isolate. The decision against surgical intervention was made after multidisciplinary evaluation by the cardiothoracic team. Conservative management was chosen based on the patient's hemodynamic stability, absence of heart failure, and rapid clinical improvement under antibiotic therapy. Close clinical and echocardiographic monitoring were ensured throughout treatment. The total duration of therapy was 6 weeks. Renal function was closely monitored throughout the course and remained stable, with eGFR ranging between 55–60 ml/min/1.73 m^2^. The clinical course was favorable, marked by defervescence, absence of new secondary infectious foci, and normalization of biological markers. Follow-up transthoracic echocardiogram at the end of treatment demonstrated complete resolution of the annular abscess with no residual vegetations. Subsequent clinical, biological, and echocardiographic assessments 1- and 3-months post-therapy confirmed sustained remission and absence of relapse. Table 1 chronologically summarizes the patient's major symptoms, diagnostic findings, interventions, and outcomes.

**Table 1:** Timeline of the patient's clinical presentation, diagnostic work-up, and management.

**Day (course)**	**Clinical event**	**Diagnostic findings**	**Management**	**Outcome**
−14 (pre-admission)	Self-limited respiratory illness	Not investigated	IM dexamethasone	Symptom resolution
−3 (pre-admission)	Acute right knee pain and swelling	Suspected septic arthritis	Referred for hospital admission	Admitted
0 (admission)	Right knee arthritis, fever	CXR: RLL consolidation; synovial fluid leukocytosis	Arthrotomy; IV cefazolin	Developed hypoxemia
2	Acute respiratory distress	CT: RLL consolidation, bilateral GGO	Oxygen supplementation	Respiratory stabilization
3–4	Acute confusional state	Brain MRI: leptomeningeal enhancement (suggestive of meningitis)	IV vancomycin added	Neurological improvement
5	Right wrist arthritis	US: synovitis with purulent drainage	Surgical drainage	Resolution of local symptoms
6–7	Persistent fever	Blood and synovial cultures: *S. pneumoniae*; TTE negative	Cefotaxime + gentamicin initiated	Afebrile but ongoing suspicion for IE
7	Anemia and coagulopathy	Laboratory profile consistent with DIC	Supportive management	Hematological stabilization
8	Suspected endocarditis	TEE: AV vegetation with annular abscess → definite IE	Cefotaxime + vancomycin on day 14	Diagnosis of Austrian syndrome established
Weeks 2–6	Continuation of antimicrobial therapy	Serial clinical and laboratory monitoring	6-week IV antimicrobial therapy	Clinical and microbiological resolution
End of therapy (day 42)	Completion of IV antibiotics	TTE: resolution of abscess and vegetations	No surgical intervention required	Sustained remission
1–3 months follow-up	Outpatient follow-up visits	Echocardiography: no relapse	Ongoing clinical monitoring	Full recovery

AV, aortic valve; CT, computed tomography; CXR, chest radiograph; DIC, disseminated intravascular coagulation; GGO, ground-glass opacities; IE, infective endocarditis; IV, intravenous; MRI, magnetic resonance imaging; RLL, right lower lobe; TEE, transoesophageal echocardiogram; TTE, transthoracic echocardiogram; US, ultrasonography.

Day 0 corresponds to the day of hospital admission. Presenting symptoms, diagnostic investigations, microbiological and imaging findings, therapeutic interventions, and clinical outcomes are presented sequentially in relation to this reference point.

## Discussion

*Streptococcus pneumoniae* remains a pathogen of considerable clinical importance due to its diverse spectrum of disease, despite the marked decline in incidence of several pneumococcal infections following the advent of effective antimicrobial therapies and widespread vaccination. Among these, pneumococcal IE has become increasingly uncommon, currently accounting for < 3% of all IE cases. Recent epidemiological data from Spanish cohorts reported incidence rates of 0.86 and 0.5%, respectively, underscoring its rarity in contemporary clinical practice (6, 7).

Nevertheless, *S. pneumoniae* continues to be a predominant etiologic agent of community-acquired pneumonia and meningitis in adults, conditions which retain high morbidity and mortality despite therapeutic advances (8). Austrian syndrome, a triad comprising pneumococcal pneumonia, meningitis, and endocarditis, represents a particularly severe and infrequent manifestation of IPD. Recognition of this syndrome necessitates heightened clinical suspicion, particularly in individuals with established risk factors such as advanced age, male sex, chronic alcohol use, and immunosuppression (8).

In the present case, the patient exhibited two recognized risk factors: advanced age and male sex. Notably, the absence of pneumococcal vaccination likely predisposed him to IPD, highlighting the ongoing relevance of vaccination strategies in mitigating the burden of severe pneumococcal infections (9). Both blood and synovial fluid isolates in our patient were identified as *S. pneumoniae* serotype 19A. This serotype is of epidemiological concern as it is frequently associated with IPD in unvaccinated adults and has been linked to reduced susceptibility to β-lactam antibiotics. The emergence and persistence of serotype 19A strains, even after widespread pneumococcal vaccination programs, have been well documented, emphasizing the need for broader serotype coverage through extended-valency vaccines (10).

Despite the absence of classical respiratory symptoms, a pulmonary origin for the infection was clinically suspected and subsequently confirmed via radiographic imaging. This underscores the necessity for proactive investigation for pulmonary foci in suspected cases of IPD, even when respiratory symptoms are not overtly apparent. The pathogenesis of Austrian syndrome typically involves hematogenous dissemination from a primary pulmonary focus, culminating in multisystem involvement due to immune system overwhelm (2, 11).

In this case, brain MRI provided crucial diagnostic support for meningitis in the absence of cerebrospinal fluid analysis. MRI is the most sensitive neuroimaging modality for detecting meningeal inflammation, with post-contrast T1-weighted and FLAIR leptomeningeal enhancement serving as reliable markers of acute bacterial meningitis. Although not specific, these findings can substantiate the diagnosis when direct microbiological confirmation is unavailable and may identify associated complications such as cerebritis or ventriculitis (12).

Pneumonia has been documented as the initial manifestation in up to 10% of immunocompetent adults who later develop septic arthritis, a pattern also observed in this case (8, 13). Pneumococcal osteoarticular infections are rare in immunocompetent individuals, comprising fewer than 20% of all cases. Among these, the knee joint is most frequently affected (38%), and involvement of multiple native joints occurs in 13.8 to 30% of cases (14, 15). Hematogenous spread remains the primary mechanism for joint infection in adults, in line with the dissemination pattern observed here (16).

Austrian syndrome itself is a rare clinical entity; only 26% of patients with pneumococcal endocarditis demonstrate the full triad of pneumonia, meningitis, and endocarditis (17). Additional involvement of other organs, particularly osteoarticular structures, is exceedingly rare, further distinguishing this case. While few reports have described Austrian syndrome associated with septic arthritis particularly in immunocompromised individuals, this remains an exceptional occurrence in immunocompetent hosts. Table 2 summarizes the demographic, clinical, and therapeutic characteristics of our patient, alongside those reported in previously published case reports.

**Table 2:** Comparative clinical and management features of the present case and previously reported cases of pneumococcal endocarditis associated with Austrian syndrome.

**Criteria**	**Our patient**	**Shin et al. (9)**	**Guerreiro et al. (4)**	**Madu et al. (8)**
Age (years)	71	43	84	62
Sex	Male	Male	Female	Male
Pneumococcal vaccination	Not vaccinated	Not specified	Not documented	Not documented
Initial respiratory symptoms	Absent	Present (fever, hypoxia)	Present (pneumonia diagnosed later)	Absent
Diagnosis of pneumonia	Yes (clinical suspicion and CT confirmation)	Yes (bilateral pneumonia)	Yes (diagnosed post-ICU admission)	Yes (diagnosed post-ICU admission)
Endocarditis presentation	Atypical	Typical (aortic murmur at admission)	Typical (mitral vegetations on TEE)	Typical (mitral vegetations on TEE)
Initial TTE	Negative	Positive (aortic vegetation)	Positive (mitral valve vegetation)	Positive (mitral valve vegetation)
Peripheral signs of endocarditis	Absent	Not specified	Not specified	Not specified
Valvular involvement	Aortic valve (partial destruction with annular abscess)	Aortic valve with abscess	Mitral valve (partial destruction)	Mitral valve (partial destruction)
Septic arthritis	Two native joints	No	Monoarthritis	No
DIC	Yes	Yes (fatal postoperative course)	Not reported	Not reported
Cardiac surgery	No	Yes (aortic valve replacement)	No (not eligible for surgery)	Yes (delayed mitral valve repair)
Antibiotic therapy	Cefotaxime + gentamicin switched to cefotaxime + vancomycin	Ceftriaxone + vancomycin	Ceftriaxone, later switched to Cefotaxime	Cefotaxime
Duration of therapy	6 weeks	Not specified	6 weeks	4 weeks
Clinical outcome	Complete recovery (medical therapy only)	Death despite surgical intervention	Death on day 7	Complete recovery (surgical and medical therapy)

CT, computed tomography; DIC, disseminated intravascular coagulation; ICU, intensive care unit; TEE, transoesophageal echocardiogram; TTE, transthoracic echocardiogram.

Reported data include patient demographics, underlying conditions, clinical presentation, diagnostic findings, management strategies, and outcomes.

Cardiac involvement in Austrian syndrome frequently affects the aortic valve, though this is not pathognomonic. The syndrome's eponym originates from Robert Austrian, who first described the preferential involvement of the aortic valve in conjunction with pneumococcal pneumonia and meningitis (3). Left-sided valves, particularly the aortic and mitral valves, are more susceptible to endocardial infection due to higher pressure gradients, turbulent flow, and pre-existing structural abnormalities. Aortic valve involvement has been documented in 44%−56% of pneumococcal endocarditis cases (2, 8).

Diagnosis of cardiac involvement is often delayed due to nonspecific clinical findings. In this case, despite an initial normal TTE, persistent clinical suspicion based on modified Duke criteria warranted TEE, which ultimately revealed aortic valve endocarditis complicated by an annular abscess. This affirms the diagnostic value of TEE and the necessity of maintaining vigilance when TTE findings are inconclusive.

Given the potential for conduction abnormalities such as atrioventricular block, the patient was monitored intensively with serial electrocardiography. The presence of an annular abscess and risk for severe cardiac sequelae justifies management in a cardiac intensive care setting (5, 18).

Although disseminated intravascular coagulation (DIC) is an infrequent complication of Austrian syndrome, it may arise from delayed diagnosis or inadequate early intervention (9). In this instance, prompt initiation of appropriate bactericidal therapy enabled early stabilization, preventing progression to hemorrhagic complications.

The antimicrobial strategy adopted in this case aligns with current ESC recommendations for pneumococcal endocarditis with CNS involvement. Cefotaxime was used as an alternative to ceftriaxone due to local formulary availability, with both agents demonstrating equivalent bactericidal activity and cerebrospinal fluid penetration against *S. pneumoniae* (5). The initial high-dose regimen was chosen to achieve therapeutic meningeal concentrations for a strain with reduced susceptibility to penicillin, followed by a standard dosing phase appropriate for endocarditis and septic arthritis management (5, 19, 20). Vancomycin was subsequently introduced because of the infection's severity, the formation of an annular abscess, and the reduced penicillin susceptibility of the pneumococcal strain, in accordance with ESC guidance recommending cephalosporins–glycopeptide combination therapy for complicated pneumococcal infections (5). The cefotaxime–vancomycin combination has also been endorsed by several authors as an effective regimen for severe invasive pneumococcal disease, including Austrian syndrome (8). The patient achieved complete clinical resolution following a 6-week antibiotic course, aligning with current guideline-based recommendations that advocate a 4–6-week treatment duration according to valvular involvement (5).

While surgical intervention is often required in pneumococcal endocarditis due to complications such as annular abscess, heart failure, and embolic phenomena, our patient responded favorably to medical therapy alone. Evidence from two systematic reviews demonstrated a marked reduction in mortality rates with surgical intervention, from 55.7 to 17% and from 57 to 10%, respectively, underscoring the critical role of timely surgery when indicated (6, 8). Nevertheless, conservative management may represent a viable alternative in carefully selected patients with non-staphylococcal infective endocarditis who exhibit a rapid improvement under targeted antimicrobial therapy, small and stable abscesses, and no evidence of heart block, severe valvular regurgitation, or prosthetic dehiscence (21). This case adds to the limited evidence supporting individualized, non-surgical therapeutic approaches for pneumococcal endocarditis when close clinical and echocardiographic monitoring is ensured.

Compared with previously published cases, the present report exhibits several distinctive features. The patient was elderly but immunocompetent and lacked classical risk factors such as alcoholism or chronic pulmonary disease. Multifocal septic arthritis constituted the initial clinical manifestation, preceding recognition of endocarditis and meningitis, an atypical chronological sequence seldom described in the literature. Both blood and synovial fluid isolates were confirmed as *Streptococcus pneumoniae* serotype 19A, a strain associated with invasive pneumococcal disease and reduced β-lactam susceptibility. Complete recovery was achieved through medical therapy alone despite the presence of an annular abscess.

This case illustrates the clinical complexity of Austrian syndrome, particularly in the context of atypical presentations without overt respiratory symptoms or clear initial imaging findings. It highlights several pivotal clinical insights and take-home messages. Foremost, it emphasizes the necessity for heightened diagnostic vigilance, even in immunocompetent patients lacking classic risk factors, as the syndrome's hallmark triad of pneumonia, meningitis, and endocarditis may manifest sequentially and subtly, making early recognition challenging. Persistent clinical suspicion should prompt advanced imaging such as TEE when initial TTE yields equivocal results. High-dose third-generation cephalosporins remain the cornerstone of therapy, with adjunctive vancomycin indicated in cases of penicillin-non-susceptible strain. Although surgery is typically indicated for annular abscesses, conservative, non-surgical management may be appropriate in carefully selected, hemodynamically stable patients demonstrating rapid clinical response under close echocardiographic surveillance. Finally, the identification of *Streptococcus pneumoniae* serotype 19A underscores both the persistence of vaccine-preventable strains and the critical need to strengthen adult immunization strategies to reduce the burden of severe pneumococcal infections such as Austrian syndrome.

## Patient perspective

In keeping with CARE guidelines, the patient perspective is presented below:

“*I am a 71-year-old man who was already dependent on others for my daily needs. A few weeks ago, I suddenly developed pain and swelling in my right knee. I thought it might just be arthritis, but the pain got worse and soon I felt very weak and feverish. When I arrived at the hospital, I started having trouble breathing and became very confused—it was a frightening experience not understanding what was happening to me. The doctors and nurses explained that I had a severe infection affecting my knee and lungs, and they also suspected it had spread to my brain and heart. I remember feeling scared, especially when they spoke of meningitis and endocarditis. They took many tests and gave me strong antibiotics and surgery on my joints. Slowly, day by day, I started to feel a bit better as the antibiotics began to work. The confusion lifted, and I could breathe normally again. The hospital staff were very caring and kept me informed, which helped me stay hopeful. Now that I have fully recovered, I am grateful for their care. I also realize that I never had the pneumonia vaccine; if I had, maybe this never would have happened. I hope sharing my story reminds other older people to stay up to date on vaccinations.”*

## Data Availability

The original contributions presented in the study are included in the article/supplementary material, further inquiries can be directed to the corresponding author.

## References

[1] Weiser JN Ferreira DM Paton JC. *Streptococcus pneumoniae*: transmission, colonization and invasion. Nat Rev Microbiol. (2018) 16:355–67. doi: 10.1038/s41579-018-0001-8 29599457 PMC5949087

[2] Dalal A Ahmad H. Austrian syndrome (pneumococcal pneumonia, meningitis, and endocarditis): a case report. Am J Med Sci. (2008) 336:354–5. doi: 10.1097/MAJ.0b013e31815bd256 18854681

[3] Austrian R. Pneumococcal endocarditis, meningitis, and rupture of the aortic valve. Arch Intern Med. (1957) 99:539–44. doi: 10.1001/archinte.1957.00260040039004 13410159

[4] Guerreiro G Monteiro AP Coelho L Póvoa P. Pneumococcus beyond an Austrian syndrome – a case report. IDCases. (2022) 28:e01486. doi: 10.1016/j.idcr.2022.e01486 35392600 PMC8980754

[5] Delgado V Ajmone Marsan N de Waha S Bonaros N Brida M Burri H . 2023 ESC guidelines for the management of endocarditis. Eur Heart J. (2023) 44:3948–4042. doi: 10.1093/eurheartj/ehad193 37622656

[6] De Egea V Muñoz P Valerio M de Alarcón A Lepe JA Miró JM . Characteristics and outcome of *Streptococcus pneumoniae* endocarditis in the XXI century: a systematic review of 111 cases (2000–2013). Medicine. (2015) 94:e1562. doi: 10.1097/MD.0000000000001562 26426629 PMC4616835

[7] Olmos C Vilacosta I Fernández-Pérez C Bernal JL Ferrera C García-Arribas D . The evolving nature of infective endocarditis in Spain. J Am Coll Cardiol. (2017) 70:2795–804. doi: 10.1016/j.jacc.2017.10.005 29191329

[8] Madu A Alex-Okoro T Okoduwa A Cotton J. Austrian syndrome: report of one case and a systematic review of case reports — new insights. Clin Med. (2024) 24:100205. doi: 10.1016/j.clinme.2024.100205 38649138 PMC11109293

[9] Shin YI Papyan N Cedeño H Stratidis J. Austrian syndrome: the deadly triad. IDCases. (2020) 22:e00948. doi: 10.1016/j.idcr.2020.e00948 32923368 PMC7473259

[10] Song JY Nahm MH Moseley MA. Clinical implications of pneumococcal serotypes: invasive disease potential, clinical presentations, and antibiotic resistance. J Korean Med Sci. (2013) 28:4–15. doi: 10.3346/jkms.2013.28.1.4 23341706 PMC3546102

[11] Battisha A Madoukh B Altibi A Sheikh O. A rare presentation of Austrian syndrome with septic arthritis in an immunocompetent female. Egypt Heart J. (2019) 71:9. doi: 10.1186/s43044-019-0010-6 31659532 PMC6821411

[12] Saberi A Roudbary SA Ghayeghran A Kazemi S Hosseininezhad M. Diagnosis of meningitis caused by pathogenic microorganisms using magnetic resonance imaging: a systematic review. Basic Clin Neurosci. (2018) 9:73–86. doi: 10.29252/nirp.bcn.9.2.73 29967667 PMC6026091

[13] Rivera K Viles D Zielonka M Izurieta C Martínez TR Menéndez D. Austrian syndrome: resurgence of an old and deadly triad. J Intensive Med. (2024) 4:261–4. doi: 10.1016/j.jointm.2023.06.006 38681791 PMC11043638

[14] Chemsi H Chadli M Sekhsokh Y. Pneumococcal arthritis in an immunocompetent adult. Pan Afr Med J. (2015) 21:139. doi: 10.11604/pamj.2015.21.139.6421 26327976 PMC4546779

[15] Bayeh NE. Pneumococcal arthritis in adults in France: a descriptive study among 15 Regional Pneumococcal Networks (RPN) (Doctoral thesis). Amiens: Université de Picardie Jules Verne; Faculté de Médecine d'Amiens (2022). Available online at: https://dumas.ccsd.cnrs.fr/dumas-03835073v1 (Accessed June 29, 2025).

[16] Shirtliff ME Mader JT. Acute septic arthritis. Clin Microbiol Rev. (2002) 15:527–44. doi: 10.1128/CMR.15.4.527-544.2002 12364368 PMC126863

[17] Mekraksakit P Elmassry M Leelaviwat N Nugent K. Invasive pneumococcal disease confirmed in five different sites including Austrian syndrome in a male patient with methamphetamine abuse. BMJ Case Rep. (2020) 13:e239718. doi: 10.1136/bcr-2020-239718 33303512 PMC7733077

[18] Périer A Puyade M Revest M Tattevin P Bernard L Lemaignen A . Prognosis of *Streptococcus pneumoniae* endocarditis in France, a multicenter observational study (2000–2015). Int J Cardiol. (2019) 288:102–6. doi: 10.1016/j.ijcard.2019.04.048 31056415

[19] Hoen B Varon E de Debroucker T Fantin B Grimprel E Wolff M . Management of acute community-acquired bacterial meningitis (excluding newborns). Long version with arguments. Med Mal Infect. (2019) 49:405–41. doi: 10.1016/j.medmal.2019.03.009 31402154

[20] Stahl JP Canouï E Pavese P Bleibtreu A Dubée V Ferry T . SPILF update on bacterial arthritis in adults and children. Infect Dis Now. (2023) 53:104694. doi: 10.1016/j.idnow.2023.104694 36948248

[21] Habib G Badano L Tribouilloy C Vilacosta I Zamorano JL Galderisi M . Recommendations for the practice of echocardiography in infective endocarditis. Eur J Echocardiogr. (2010) 11:202–19. doi: 10.1093/ejechocard/jeq004 20223755

